# DNA methylation profile in patients with indolent systemic mastocytosis

**DOI:** 10.1002/clt2.12074

**Published:** 2021-11-02

**Authors:** Aleksandra Górska, Ewa Jabłońska, Edyta Reszka, Marek Niedoszytko, Magdalena Lange, Marta Gruchała‐Niedoszytko, Justyna Jarczak, Dominik Strapagiel, Magdalena Górska‐Ponikowska, Paulina Bastian, Iwona Pelikant‐Małecka, Leszek Kalinowski, Bogusław Nedoszytko

**Affiliations:** ^1^ Department of Allergology Medical University of Gdansk Gdansk Poland; ^2^ Department of Translational Research Nofer Institute of Occupational Medicine Lodz Poland; ^3^ Department of Dermatology, Venerology and Allergology Medical University of Gdansk Gdansk Poland; ^4^ Department of Clinical Nutrition Medical University of Gdansk Gdansk Poland; ^5^ Department of Molecular Biophysics University of Lodz Lodz Poland; ^6^ Department of Medical Chemistry Medical University of Gdansk Gdansk Poland; ^7^ Department of Clinical Analytics Medical University of Gdansk Gdansk Poland; ^8^ Invicta Fertility and Reproductive Center Molecular Laboratory Sopot Poland

**Keywords:** DNA methylation, epigenetics, KIT mutation, mastocytosis

## Abstract

**Background:**

Mastocytosis is a clinically heterogeneous, usually acquired disease of the mast cells with a survival time that depends on the onset of the disease and ranges from skin‐limited to systemic disease, including indolent and more aggressive variants. The crucial element in pathogenesis is the presence of oncogenic KIT somatic mutation D816V. Further epigenetic alterations are responsible for regulating the expression of genes. It is essential to identify indicators of disease progression, and the specific clinical picture to establish an appropriate therapeutic strategy.

**Objective:**

The aim of this study was to analyze the relation of mastocytosis symptoms and epigenetic changes, and to identify epigenetic predictors of the disease.

**Methods:**

Global DNA methylation profile analysis was performed in peripheral blood collected from 73 patients with indolent systemic mastocytosis (ISM) and 43 healthy adult volunteers. Levels of 5‐methylcytosine (5‐mC) and 5‐hydroxymethylcytosine (5‐hmC) were determined using an ELISA‐based method, while the methylation of the Alu and LINE‐1 repeats were assayed with the quantitative methylation‐specific PCR technique. A questionnaire interview was conducted among the study participants to collect data on possible epigenetic modifiers. Additionally, the methylation profile was compared between three human mast cell lines: ROSA KIT D816V, ROSA KIT WT, and HMC‐1.1 KIT V560G, in order to assess the association between *KIT* mutations and methylation profile.

**Results:**

A significantly lower level of DNA hydroxymethylation (5‐hmC) in the blood was found in patients with ISM as compared to the controls (0.022% vs. 0.042%, *p* = 0.0001). Differences in the markers of global DNA methylation (5‐mC, Alu, LINE‐1) were not statistically significant, although they did indicate generally higher DNA methylation in patients with mastocytosis. The 5‐hmC level was significantly associated with allergy (*p* = 0.011) in patients with ISM, showing a higher level of 5‐hmC in patients with allergy as compared to patients without allergy. The in vitro study revealed significant differences between the studied cell lines at the level of 5‐mC, Alu, and LINE‐1.

**Conclusions:**

This study confirms that epigenetic changes are involved in mastocytosis, and suggests that allergy may be an important epigenetic modifier of the disease. A possible association between *KIT* mutations and methylation status observed in human mast cell lines requires further investigation in human studies.

**Clinical Implications:**

Epigenetic alterations are involved in mastocytosis pathology. The possible role of allergy as an important epigenetic modifier suggests the more impaired function of mast cells in ISM patients without allergy.

**Capsule summary:**

Decreased DNA demethylation in the blood DNA of patients with ISM confirms that epigenetic alterations are involved in mastocytosis pathology. We observed a possible role of allergy as an important epigenetic modifier. There is a possible association between KIT mutations and the methylation status observed in human mast cell lines.

## 1 INTRODUCTION

Mastocytosis is a unique hematologic neoplasm, characterized by an expansion and accumulation of atypical mast cells (MCs) in different organs including bone marrow, skin, liver, spleen, lymph nodes, and gastrointestinal tract. The course of the disease depends on the subvariant diagnosis, clinical presentation, and outcome as well as comorbidities.^1^ Based on the classification of the World Health Organization (WHO), mastocytosis can be divided into subvariants of cutaneous mastocytosis (CM), systemic mastocytosis (SM), and localized MC sarcoma.^2^ The prognosis in mastocytosis is variable, from favorable in CM, not affecting the life expectancy however significantly decreasing quality of life, low progression rate in indolent systemic mastocytosis (ISM), to rapidly deteriorating aggressive forms of the disease such as aggressive systemic mastocytosis (ASM) and mast cell leukemia (MCL). The prognosis may change with disease progression or the effects of treatment. Various therapeutic targets have been reported in neoplastic MCs recently, and several of these target molecules have been the focus of clinical trials recently.^2^

The presence of oncogenic *KIT* mutation D816V is an important element in pathogenesis of mastocytosis as it has been detected in more than 80% of adult cases with SM.^3^ In pediatric patients, mainly with CM, 25% of cases have no mutation, 35% have a KIT (D816V, D816I or D816Y) mutation, and 40% have other mutations coding the extracellular part of the SCF (stem cell factor) receptor.^4^ In patients with advanced SM, mostly in aggressive SM (ASM), SM with an associated hematologic neoplasm (SM‐AHN), or MC leukemia (MCL), additional genetic changes have been identified: mutations in *TET2*, *SRSF2*, *ASXL1*, or *RUNX1*, which might play a crucial role in the development of the variety of clinical outcomes and the aggressiveness of SM, and may represent new therapeutic targets.^3^

So far, little is known about the epigenetic factors leading to the onset or progression of mastocytosis. It has been reported that the pathogenesis of mastocytosis may be influenced by abnormalities in the regulation of epigenetic mechanisms of gene expression through: specific microRNA expression, loss of suppressor gene function, activation of specific oncogenes (tyrosine kinases, signal transduction proteins), impaired DNA replication and repair processes, apoptosis, and by causing instability of the MC genome.^5^, ^6^, ^7^ A number of apoptosis‐associated genes, including *FAS* and several TSGs, including *p16* and *p21*, were found to be hypermethylated in mast cell lines HMC‐1 but not in normal bone marrow cells. Moreover, p16 and FAS were found to be hypermethylated in both HMC‐1 subclones, whereas p21 was found to be hypermethylated only in HMC‐1.2 cells harboring KIT D816V, but not in HMC‐1.1 cells lacking KIT D816V.^8^ Aberrant expression of micro RNAs is detected in mast cells with a *KIT* mutation. It has been indicated that cells with *KIT* mutations have lower expression of miR −539 and miR −381.^6^ These miRNAs are involved in the inhibition of microphthalmia‐associated transcription factor (MITF) expression which regulates the development of MC and melanocyte, and the synthesis of tryptase and melanin.^5^, ^6^, ^7^

Epigenetic alterations are responsible for regulating the expression of genes that do not modify the DNA sequence. The best‐known epigenetic marker is methylation of DNA which predominantly occurs in cytosines that precede guanines; these are called dinucleotide CpGs and are one of the epigenetic modifications frequently observed in a variety of biological and pathological processes.^9^ DNA methyltransferases (DNMTs) are involved in the process of DNA methylation by catalyzing the transfer of a methyl group to the 5‐position of the cytosine in DNA and the generation of 5‐methylcytosine (5‐mC).^10^ In the process of DNA demethylation, 5‐mC can be converted into 5‐hydroxymethylcytosine (5‐hmC) by TET hydroxylases, which play a key role in the active demethylation of DNA. Therefore, global DNA hypomethylation leads to chromosomal instability which may result in cell proliferation and cancer.^11^ There are known non‐LTR retrotransposons—including long interspersed elements—LINE‐1 and short interspersed elements—Alu—which affect the human genome in many different ways: generating insertion mutations, genomic instability, alterations in gene expression and also contributing to genetic innovation.^12^ About 1.4 million Alu of repetitive elements and 0.5 million interspersed nucleotide elements (LINE‐1 elements) in the human genome are normally heavily methylated. It is estimated that more than one‐third of DNA methylation occurs in repetitive elements. Thus, LINE‐1 and Alu methylation has been measured as a surrogate marker for global DNA methylation.^13^ Based on recent reports concerning studies on the sequencing of multiple genes for typical mutations identified in malignant myeloid disorders, a list of somatic mutations often found in patients with mastocytosis has been created.^7^, ^14^, ^15^ These genes encode factors that regulate splicing process, signaling transmission, and epigenetic regulations. The most frequently mutated genes are *TET‐2* (demethylation of DNA), *DNMT3A* (methylation of CpG islands), *ASXL1* (chromatin silencing and remodeling), and *IDH2* (regulation of histone methylation). Importantly, mutations in genes encoding factors involved in the epigenetic process are frequently observed in the more advanced form of mastocytosis, and are associated with poor prognosis and shorter life expectancy.^7^, ^14^, ^15^ A previous study has shown the antineoplastic effects of demethylating agents on human neoplastic MCs, and described that the death regulator FAS is hypermethylated in neoplastic MCs, and that 5‐azacytidine and decitabine can induce demethylation and thus re‐expression of FAS.^8^

However, so far, little is known about the epigenetic factors leading to the clinical onset of mastocytosis. In this study, we investigated the relation of mastocytosis symptoms and epigenetic changes. Secondly, we tried to identify epigenetic predictors of the disease.

## 2 METHODS

### 2.1 Case–control study

The study comprised 73 patients with ISM which were included in our local registry and treated in the Allergology Department in the years 2012–2020. Mastocytosis was diagnosed in accordance with WHO guidelines, including pathological examination of a bone marrow biopsy, examination of the bone marrow aspirate: cytology, immunophenotyping of mast cell expression of CD 2 and CD 25, activating point mutation of KIT, and serum tryptase level.^2^ Molecular analysis towards the c.2447A>T variant (p. (Asp816Val)) in the KIT gene was performed using the qPCR in bone marrow aspirate.^16^ Our local registry is part of the European Competence Network on Mastocytosis (ECNM) Registry which was described previously.^17^ The control group included 43 healthy adult volunteers who were not diagnosed with any chronic diseases including allergy. Peripheral blood samples were collected from patients with ISM during diagnosis or during the follow‐up visit in the Allergology Department, or from healthy volunteers recruited in the University of Lodz. Informed consent was obtained from all study participants. The database of the ECNM registry, data storage, and data distribution comply with the rules and regulations of data protection laws, with local ethics committee regulations of each participating center, and with the declaration of Helsinki.^17^ The enrolment to the study was performed between January and December 2019. The study was approved by the institutional review board Gdańsk: NKBBN/270/2018.

The study participants were asked to complete interview questionnaires. The interview questionnaire was created for the purposes of the research and included questions about exposure to factors potentially affecting the epigenome, including age, BMI, sex, smoking status, and regular use of medicines. Patients treated with medications which could significantly modify the methylation profile (as oral glucocorticoids, immunosuppressive and oncologic drugs) were excluded from the analysis. Data on the tryptase serum concentration and symptoms of allergy or anaphylaxis were collected in the patient group from their medical records.

Assessment of hypersensitivity to pollen, foods, drugs, insect venom, and latex was based on clinical symptoms. Further, the diagnosis of allergy was confirmed by skin prick tests and/or specific IgE evaluation. Insect venom allergy was diagnosed in accordance with EAACI guidelines, and included specific IgE evaluation and both skin and intracutaneous tests in all patients in concordance with the symptoms of insect venom allergy in their medical history. Drug hypersensitivity (non‐steroid anti‐inflammatory drugs, antibiotics, local anesthetics) was diagnosed in accordance with EAACI/ENDA guidelines, and was confirmed by both skin prick and/or intracutaneous tests which were followed by the drug provocation tests (DPT) in a selected group of patient.^18^

### 2.2 Cell study

ROSA KIT D816V, ROSA KIT WT, and HMC‐1.1 mast cell lines were cultured in an incubator at 37°C and 5% CO_2_ for 48 h. The composition of the cellular medium was dependent on the type of cell line. HMC 1.1 cells were cultured in dedicated Iscove's Modified Dulbecco's Medium (Merck Millipore) supplemented with 1.2 mM of α‐thioglycerol, and fetal bovine serum (FBS) so that its final concentration was 10%, and antibiotics (up to 1%). The medium for ROSA KIT D816V consisted of Iscove's Modified Dulbecco's Medium (Merck Millipore) with FBS (up to 10%) and antibiotics (up to 1%). While the medium for ROSA KIT WT was also supplemented with rhSCF Stem Cell Growth Factor (R&D Systems) at a final concentration of 80 ng/ml. All cell lines were incubated in triplicates.

### 2.3 DNA methylation analysis

Genomic DNA was isolated from whole blood and cell lysates (10^6^ cells) using a commercial kit (HP Template PREPARATION Kit, Roche) in order to assess the methylation profile. The amount of the total extracted DNA was determined in microplates using the spectrophotometric method (Multi Scan Go, Thermo Fisher).

5‐mC and 5‐hmC levels in 100 ng of genomic DNA were quantified with a MethylFlash Global DNA Methylation (5mC) ELISA Easy Kit, and a MethylFlash Global DNA Hydroxymethylation (5‐hmC) ELISA Easy Kit according to the manufacturer's instructions (Epigentec).

Methylation of Alu and LINE‐1 was performed by the use of the MethyLight methodology.^19^ Briefly, 500 ng of DNA from each sample was independently treated with sodium bisulfite using an EZ DNA Methylation‐Gold™ kit (Zymo Research, Irvine, California, CA, USA) in the presence of CpG methylated Jurkat genomic DNA (a positive control, fully methylated) and 5‐Azadc‐treated unmethylated Jurkat genomic DNA (a negative control) (NEB, Ipswich, MA, US). The methylation status of the Alu and LINE‐1 CpG sites was assessed using the quantitative methylation‐specific PCR (QMSP) technique with MethyLight fluorescent probes with a set of primers complementary to the methylated sequence^20^ using a FastStart Essential DNA Probes Master (Roche). ACTB was used: forward 5′‐TGGTGATGGAGGAGGTTTAGTAAGT‐3′, reverse 5′‐AACCAATAA‐AACCTACTCCTCCCTTAA‐3′, probe 6FAM5′‐ACCACCACCCAA‐CACACAATAACAAACACA‐3′BHQ1, as a reference gene without CpG sites to control for DNA input.^21^

Based on the Dfam2 database with information on repetitive DNA families (https://dfam.org), the methylated Alu sequence (89bp) with 7 CpG sites derives from the AluSx subfamily.^22^ LINE‐1 methylated sequence (81bp) possesses 9 CpG sites (GenBank accession number X52235, https://ncbi.nlm.nih.gov).

### 2.4 Statistical analysis

Data were analyzed using the Statistica 13.0 software (Stat‐Soft Inc). Group differences were assessed with the Mann–Whitney *U* test or the Kruskal–Wallis test followed by the post‐hoc Dunn's test. Spearman's rank correlation coefficients were used to describe the correlations between the variables. *p* values below 0.05 were considered to be statistically significant.

## 3 RESULTS

The study group characteristics are presented in Table 1. The group of cases comprised 73 adult patients with ISM, including 50 women (68.5%) and 23 men (31.5%), with a median age of 50.2 years, and with a median BMI value of 26.9. The control group consisted of 43 healthy adults including 21 women (48.8%) and 22 men (51.2%), with a median age of 39.2 years and with a median BMI value of 24.7. The male‐to‐female ratio was smaller in the group of patients as compared to controls (*p* = 0.036). Both groups were significantly different in terms of age (*p* < 0.0001) and BMI (*p* = *0.013*). Most of the patients and controls (76.7% and 76.7%, respectively) declared themselves as non‐smokers. The majority of the patients were under medical treatment, which concerned mainly regular use of class H1‐ and/or H2‐receptor antagonists. KIT D816V was detected in 86.8% (59/68; *N* = 68) of the ISM cases. The presence of the KIT D816V mutation was found in 75% (*N* = 18) of patients with anaphylaxis, and 92% (*N* = 39) of patients without anaphylaxis (*p* = 0.042). For two patients with KIT D816V no data were obtained regarding neither symptoms of anaphylaxis nor allergy. Patients with concomitant allergy (*n* = 36, 49.3%) presented symptoms in reaction to: Hymenoptera venom (*n* = 19, 52.8%), drugs (*n* = 10, 27.8%), food (*n* = 4, 11.1%), aeroallergens (*n* = 3, 8.3%), and latex (*n* = 1, 2.8%). Symptoms of anaphylactic reaction (*n* = 25, 34.2%) were triggered by insect stings (*n* = 19, 76%), drugs (*n* = 4, 16%), and unknown trigger (*n* = 2, 8%).

**TABLE 1:** Study group characteristics

	ISM (*n* = 73)	Controls (*n* = 43)	*p*
Age (years)	50.2 (20–75)	39.2 (23–69)	*p* < 0.0001^a^
BMI (kg/m^2^)	26.9 (17–40.8)	24.7 (17.3–30.5)	*p* = 0.013^a^
Sex			
Males, *n* (%)	23 (31.5)	22 (51.2)	
Females, *n* (%)	50 (68.5)	21 (48.8)	*p* = 0.036^b^
Smoking status			
Yes, *n* (%)	14 (19.2)	6 (14.0)	
No, *n* (%)	56 (76.7)	33 (76.7)	
No data, *n* (%)	3 (4.1)	4 (9.3)	
Medication			
Yes, *n* (%)	63 (86.3)	9 (20.9)	
No, *n* (%)	7 (6.6)	20 (46.5)	
No data, *n* (%)	3 (4.1)	14 (32.6)	
Tryptase (μg/L)	38	Data not collected	
	6.5–263		
Allergy			
Yes, *n* (%)	36 (49.3)	Data not collected	
No, *n* (%)	35 (47.9)		
Anaphylaxis			
Yes, *n* (%)	25 (34.2)	Data not collected	
No, *n* (%)	46 (63)		
KIT D816V			
Yes, *n* (%)	59 (86.8)	Data not collected	
No, *n* (%)	9 (12.3)		
No data, *n* (%)	5 (6.8)		

*Note*: Data for age, BMI and tryptase serum concentration are expressed as median and range.

Abbreviation: ISM, indolent systemic mastocytosis.

^a^ Mann–Whitney *U* test.

^b^ Chi‐Square test.

**FIGURE 1:**
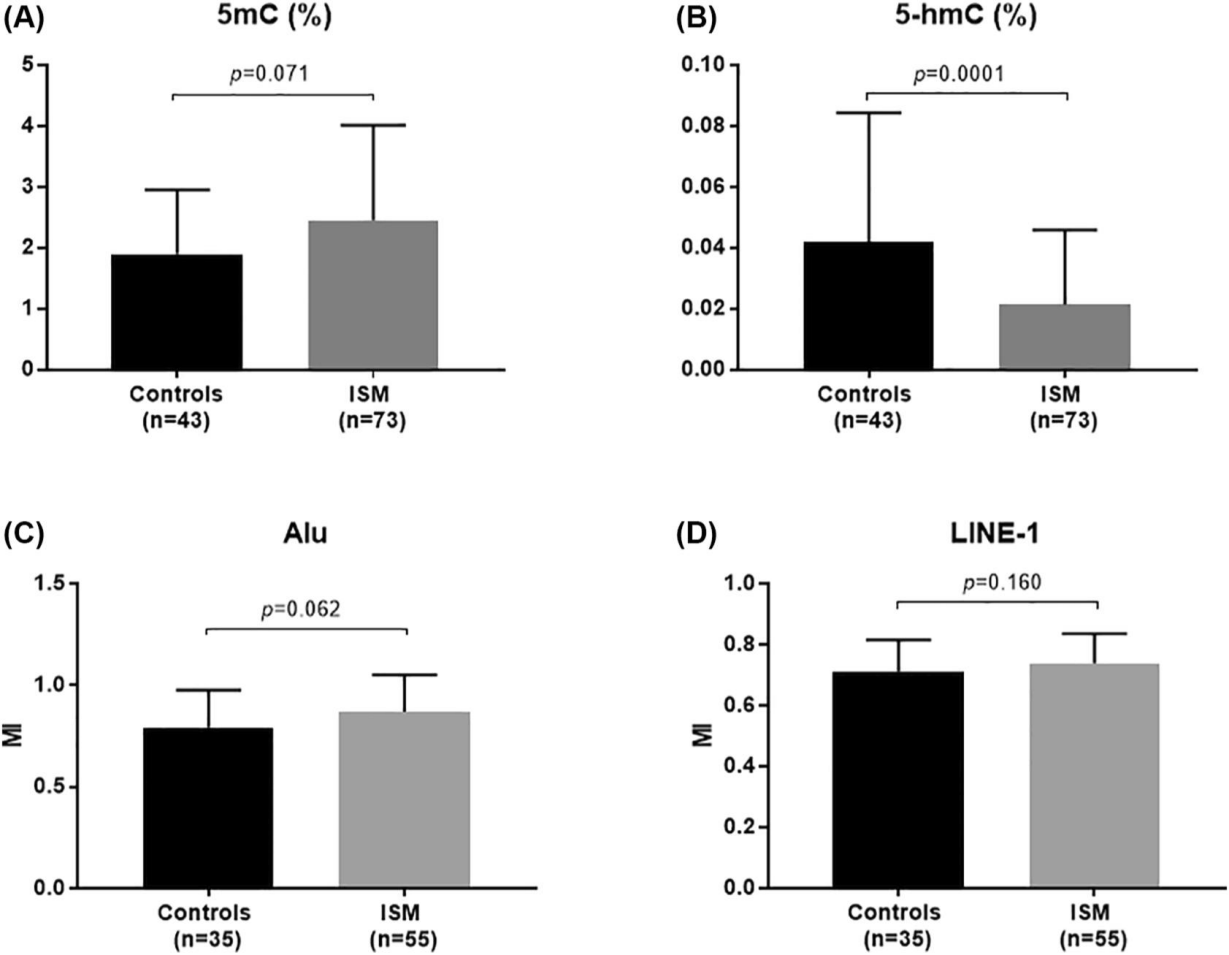
Markers of blood DNA methylation: (A) 5‐mC of total DNA, (C) Alu, (D) LINE‐1, and demethylation: (B) 5‐hmC of total DNA, in patients with indolent systemic mastocytosis and control group

**FIGURE 2:**
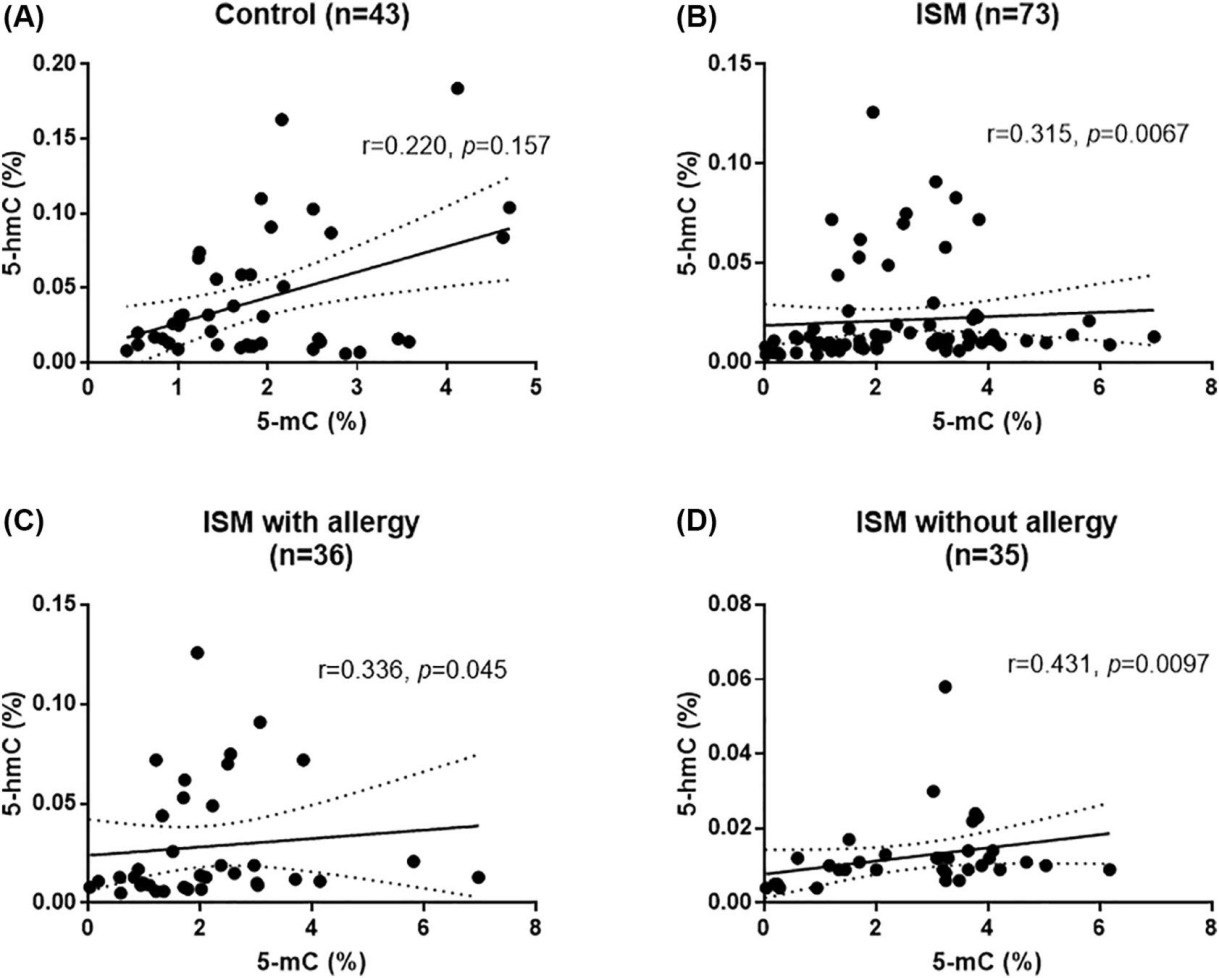
Correlation of global DNA methylation (5‐mC of total DNA) and hydroxymethylation (5‐hmC of total DNA) in the control group (A), ISM patients (B), ISM patients with allergy (C), and ISM patients without allergy (D). For two patients, data on allergy symptoms were not available

The 5‐mC and 5‐hmC levels were successfully determined in 116 (100%) collected samples. For Alu and LINE‐1, 90 samples (35 cases and 55 controls) were selected for analysis based on DNA concentration, modified with sodium bisulfite. The group differences at the level of methylation and demethylation markers (5‐mC, 5‐hmC, Alu and LINE‐1) are presented on Figures 1 and 2. A significantly lower level of DNA hydroxymethylation (5‐hmC) in blood DNA was found in the patients with ISM as compared to the controls (0.022 vs. 0.042, *p* = 0.0001, Figure 1). Differences in the markers of global DNA methylation (5‐mC, Alu, LINE‐1) were not statistically significant (Figures 1 and 2), although they did indicate generally higher DNA methylation in patients with mastocytosis.

In the next stage in our research, we analyzed the possible association of methylation/demethylation markers with individual factors in both study groups, as presented in Table 2.

**TABLE 2:** Association of markers of DNA methylation and demethylation with individual factors in patients with ISM and control group

	Markers of methylation and demethylation							
	ISM (*n* = 73)				Controls (*n* = 43)			
Factor	5‐mC	5‐hmC	Alu	LINE‐1	5‐mC	5‐hmC	Alu	LINE‐1
Age^a^	*p* = 0.945	*p* = 0.063	*p* = 0.729	*p* = 0.807	*p* = 0.291	*p* = 0.381	*p* = 0.853	*p* = 0.892
BMI^a^	*p* = 0.575	*p* = 0.618	*p* = 0.678	*p* = 0.587	*p* = 0.541	*p* = 0.429	***p = *0.034**	*p* = 0.153
Sex^b^	*p* = 0.789	*p* = 0.472	*p* = 0.799	*p* = 0.978	*p* = 0.688	*p* = 0.990	*p* = 0.398	*p* = 0.679
Smoking status^b^	*p* = 0.424	*p* = 0.063	*p* = 0.762	*p* = 0.712	*p* = 0.741	*p* = 0.267	*p* = 0.188	*p* = 0.436
Medication^b^	*p* = 0.281	*p* = 0.273	*p* = 0.243	*p* = 0.503	*p* = 0.248	*p* = 0.358	*p* = 0.121	*p* = 0.832
Tryptase (μg/ml)^a^	*p* = 0.689	*p* = 0.622	*p* = 0.771	*p* = 0.545	Data not collected			
Allergy^b^	*p* = 0.057	***p* = 0.011**	*p* = 0.987	*p* = 0.390				
Anaphylaxis^b^	*p* = 0.240	*p* = 0.140	*p* = 0.579	*p* = 0.870				
KIT D816V	*p* = 0.055	*p* = 0.704	*p* = 0.862	*p* = 0.079				

*Note*: Significant *p* values are in bold.

Abbreviation: ISM, indolent systemic mastocytosis.

^a^ Spearman's correlation coefficient.

^b^ Mann–Whitney *U* test.

Age, sex, and BMI were not correlated with any of the analyzed markers of DNA methylation or demethylation, apart from significant correlation between Alu and BMI in the control group (*r* = 0.376, *p* = 0.034).

A significant effect of allergy on the 5‐mC and 5‐hmC levels in the patients with mastocytosis was observed. The trend of lower levels of 5‐mC (of borderline significance, *p* = 0.057) and significantly higher levels of 5‐hmC (*p* = 0.011) were observed in patients with allergic symptoms compared to patients without allergy (Figure S1). No significant differences were found at the level of DNA methylation or demethylation with respect to the KIT D816V mutation; however, comparison was limited by the small sample size of the group without mutation (nine subjects). Nevertheless, the trend of a higher level of DNA methylation in KIT D816V carriers was noted.

A statistically significant and positive correlation between 5‐mC and 5‐hmC (*r* = 0.315, *p* = 0.0067, Figure 2B) was found in the patients with ISM. In the control group, the correlation between 5‐mC and 5‐hmC was positive, though not significant (*r* = 0.220, *p* = 0.157, Figure 2A). The group of patients was not homogeneous in terms of epigenetic phenotype, showing a distinct group of subjects with higher values of 5‐hmC and lower values of 5‐mC. Therefore, an additional analysis was carried out, grouping the patients according to the presence of allergy (as a factor influencing the level of 5‐hmC), which showed that the group of distinct epigenetic phenotype (high 5‐hmC accompanied with low 5‐mC level) concerned mainly patients with allergy symptoms (Figure 2C). Compared to the patients with allergy, the patients without symptoms of allergy (Figure 2D) were characterized by a stronger correlation between the 5‐mC and 5‐hmC level (*r* = 0.431, *p* = 0.0097). Clearly, the subgroup of patients with allergy and distinct epigenetic phenotype weakened the overall correlation between 5‐mC and 5‐hmC in the entire group of patients.

**FIGURE 3:**
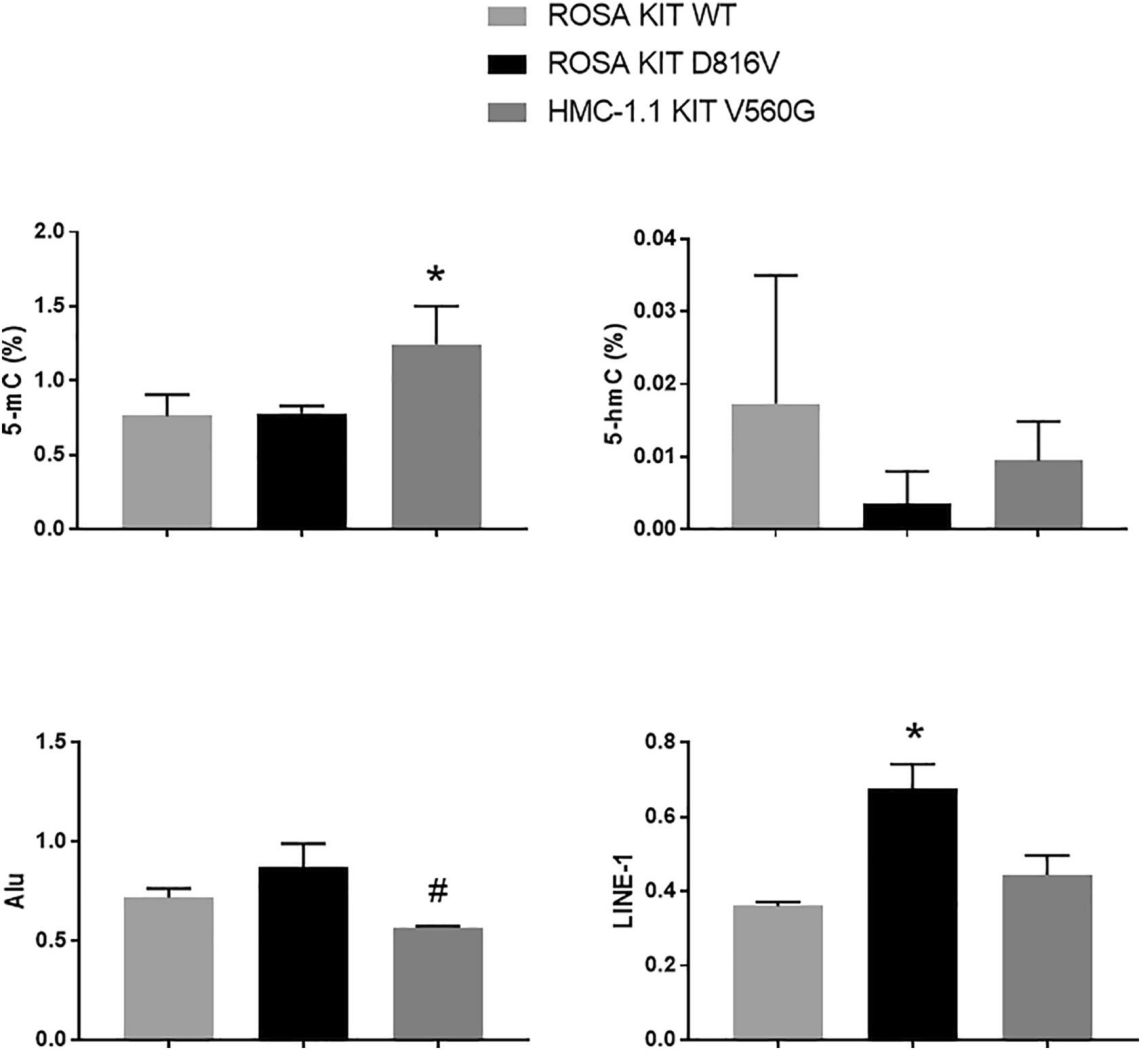
Level of DNA methylation/demethylation in three human mast cell lines with significant differences indicated by a multiple comparison test following the Kruskal–Wallis test. *p < 0.05 as compared to ROSA KIT WT. ^#^ *p* < 0.05 as compared to ROSA KIT D816V

The comparative DNA methylation analysis performed in the human mast cell lines revealed significant differences at the level of 5‐mC and LINE‐1, pointing generally to higher DNA methylation in cells harboring *KIT* mutations. Conversely, 5‐hmC was observed to be non‐significantly lower in the lines with *KIT* mutations as compared to the wild‐type line, ROSA KIT WT (Figure 3).

## 4 DISCUSSION

In this study, we found a significantly lower 5‐hmC content in the blood DNA of patients with mastocytosis compared to healthy controls. We also found lower but not significant levels of 5‐mC, and simultaneously significantly higher levels of 5‐hmC in patients with allergic symptoms compared to patients without allergies, which suggests that allergy is associated with DNA demethylation in mastocytosis and thus may be an important factor involved in the epigenetic changes in this disease. Additionally, the cell study suggests that KIT mutations may be associated with epigenetic alterations, leading to increased DNA methylation and possibly decreased demethylation.

Epigenetic alterations are responsible for regulating the expression of genes. Different levels of epigenetic regulation are described, including DNA methylation, post‐transcriptional modifications by non‐coding RNAs (ncRNAs), histone modifications, and nucleosome positioning, consequently affecting DNA and chromatin structure, respectively.^23^ A correlation between increased tumor risk or severity with the mutated DNA‐modifying enzymes DNMT3A and TET2 has been observed.^24^, ^25^ Importantly global DNA hypo‐ and hypermethylation of specific tumor‐suppressor genes have been reported in tumors and in normal tissue of patients. Moreover epigenetic alterations may precede tumorigenesis or appear early in the cancer process.^11^, ^26^ The impact of epigenetic regulation on mast cells seems certain, and moreover TET2 mutations were found in 15%–29% of patients with SM, and correlated with overall worse survival.^27^, ^28^ Thus both 5‐mC and 5‐hmC might be involved in many biological processes such as gene‐control mechanisms, and regulation of DNA methylation, and may be observed in many diseases, especially in hematologic malignancies.^10^, ^25^ The interrelation of 5‐mc with 5‐hmC was already described in the 90s, and demonstrated in in vitro studies in normal DNA as a response to oxidative stress.^29^ 5‐hmC levels are decreased in various malignancies, including glioblastoma, melanoma, breast, prostate, hepatic, gastric, and renal cancers.^30^ Moreover, the loss of 5‐hmC or decreased levels of 5‐hmC are associated with poorer outcomes in melanoma, and the progressive loss of 5‐hmC was revealed in the epigenome from begin nevus to malignant melanoma.^31^ Thus, decreased levels of 5‐hmC are assumed to be an epigenetic hallmark of melanoma.^31^ A low 5‐hmC level is also associated with poor prognosis in renal cell carcinoma, gastric, hepatic and ovarian cancers.^26^, ^30^, ^32^, ^33^, ^34^ The significant association between low 5‐hmC levels and lymph node metastasis and an advanced stage in TNM classification in various cancers has been revealed in a meta‐analysis by Chen et al.^30^

Promoter hypermethylation and genomic hypomethylation have frequently been described in hematologic malignancies.^35^ We investigated levels of DNA methylation and hydroxymethylation specifically in patients with ISM and found a significantly lower 5‐hmC content in patients with mastocytosis compared to healthy controls. These results are consistent with results previously described by Leoni,^26^ where overall 5‐hmC levels were reduced in all patients with SM. It is also noticeable in that study that the reduction in 5‐hmC correlated with the presence of highly detectable levels of KIT D816V mutation. These results may suggest that the initial change in the DNA epigenome correlates with the appearance of the KIT D816V mutation, which may be followed by further genetic and epigenetic alterations that may account for disease variants and progression.^26^ In ISM, gene expression profiles were found to be different between patients with a history of IVA and those without.^36^ Gene ontology analysis revealed that the differentially expressed genes were involved in several pathways that regulate the balance between proliferation versus terminal differentiation: Wnt signaling pathway, focal and cell adhesion, calcium signaling, extracellular matrix interactions, pathways in cancer, and MAPK signaling. This may suggest that the pattern of gene expression related to neoplastic differentiation is more prevalent in patients without a history of allergy.^36^ Therefore, based on this study, it can be assumed that patients without a history of allergy have more pronounced mast cell dysfunction. This is consistent with our study, where we found that patients with ISM without allergies have decreased levels of demethylation markers, and therefore we can assume that the function of their mast cells is more impaired.

It is well‐known that atopic diseases develop in genetically predisposed individuals. A dramatic increase in the prevalence of allergy has been noticed over the past few decades. The prevalence of ISM in the adult population is at least 13 cases per 100,000 inhabitants, which was described in the Netherlands. The actual prevalence might be even higher as the diagnosis of ISM can be easily overlooked due to subtle symptoms and a lack of awareness of the disease.^37^ The increase in the incidence of allergic disorders may be explained by exposure to negative (risk exacerbating) environmental modifiers, and decreased exposure to positive (protective or risk‐reducing) environmental factors, or both. Environmental factors and lifestyle are considered to be key factors in the initiation of Th2 inflammation and the development of atopy and allergies.^38^ Important risk factors for the development and maintenance of allergic diseases, such as smoking, allergen exposure levels, ozone, diesel exhaust particles (DEPs), SO_2_, NO_2_, and bacterial superinfections, have been identified.^39^, ^40^ Also, repeated viral infections of the lower respiratory system, especially in the early life, may favor the development of atopic asthma and persist until adulthood.^41^ It seems possible to differentiate patients with allergic diseases from controls using their DNA methylation profile in the relevant tissue. A study on seasonal allergic rhinitis (SAR) revealed that DNA methylation profiles clearly and robustly distinguished SAR patients from controls, both during and outside the pollen season, however gene expression profiles of the same samples failed to separate patients and controls. These differences of DNA methylation were also observed in an in vitro model in which purified PBMCs from patients and healthy controls were challenged with allergen. The researchers also found changes in the proportions of memory T‐cell populations between the SAR patients and controls, and they suggested it as a possible cause of the observed difference in DNA methylation.^42^ The impact of air pollution in particular on the increased risk of developing allergic diseases, and the effects, are at least partially epigenetic. Higher average exposure to polycyclic aromatic hydrocarbons is associated with higher DNA methylation at several CpG sites on the *FOXP3* locus of peripheral blood mononuclear cells (PBMCs), and Treg cells, which are observed more clearly in children with asthma than in children without asthma.^43^ The results of a study by Somineni et al. suggest a possible role of TET1 methylation in asthmatic patients. The study revealed the loss of methylation at a single CpG site in the TET1 gene promoter and increased global 5‐hmC levels which were significantly associated with asthma. In contrast, traffic‐related air pollution (TRAP) exposure at participants' current homes significantly increased methylation at the same site.^44^ It is well known that DNA hypermethylation in the 5′ region of FOXP3 is associated with higher exposure of diesel exhaust exposure, and furthermore children with DNA hypermethylation in *FOXP3* are assumed to be at higher risk of asthma, persistent wheezing, and early transient wheezing.^45^ Other studies showed the correlation of fine particle air pollutant exposure with DNA methylation of several CpG sites in nitric oxide synthase genes in children,^46^ and a relationship between indoor exposure to NO_2_ and severe asthma in children with high ADRB2 methylation levels.^47^

We assessed the influence of clinical parameters in patients with mastocytosis on their DNA methylation profile. We did not find a correlation between age, sex, or BMI with any of the analyzed markers of DNA methylation or demethylation in patients with mastocytosis, but we revealed a significant correlation between Alu and BMI in the control group. We also found lower but not significant levels of 5‐mC, and simultaneously significantly higher levels of 5‐hmC, in patients with allergic symptoms compared to patients without allergies. Moreover, we analyzed the correlation between the 5‐mC methylation marker and 5‐hmC demethylation in the study group. As a result, a positive correlation was confirmed in the control (although not significant), but in the group of patients, there was also a positive and significant correlation. Importantly, the group of patients was not homogeneous in this respect; that is, a group of patients with high 5‐mC values, and one with low 5‐mC values was clearly visible. Therefore, an additional analysis was performed grouping the patients in terms of the presence of allergy, where a significant relationship was maintained only in the patients without allergy (*p* = 0.011).

This phenomenon may indicate an imbalance between DNA methylation and demethylation processes in patients with mastocytosis and allergy, and point toward the existence of epigenetic alterations which precede or arise from mastocytosis.

The results of our study have a few limitations including the statistical differences in the age, gender, and BMI of the controls and mastocytosis patients. However, both populations were similar in terms of age and BMI, with a similar range, and clinical conditions with no additional diseases. We plan to replicate the results in a further study in a larger, independent population.

In conclusion, this study demonstrated decreased DNA demethylation in the blood DNA of patients with ISM. The presented data may suggest a role or involvement of DNA methylation in the pathology of mastocytosis but these results should be confirmed in a larger, independent population. Moreover, the observed possible role of allergy as an important epigenetic modifier suggests that the function of mast cells in ISM patients without allergy symptoms may be more impaired due to epigenetic changes, which requires further studies. The possible association between *KIT* mutations and methylation status that was observed in human mast cell lines requires further investigation in human studies.

## CONFLICT OF INTERESTS

The funder of the study had neither a role in the design of the study, the collection, analysis, and interpretation of the data, nor in writing the manuscript. The corresponding author had full access to the results of the study and to the ECNM dataset. The corresponding author has final responsibility for all data and the content of the paper, and for the decision to submit it for publication. The corresponding author declare no conflict of interest regarding this publication.

## Supporting information

Supplementary Material 1
