# Supplementary figures and images for: DNA methylation profile in patients with indolent systemic mastocytosis
Source: Clin Transl Allergy. 2021 Nov 2;11(9):e12074. doi: 10.1002/clt2.12074 (PMC8561632; doi:10.1002/clt2.12074)

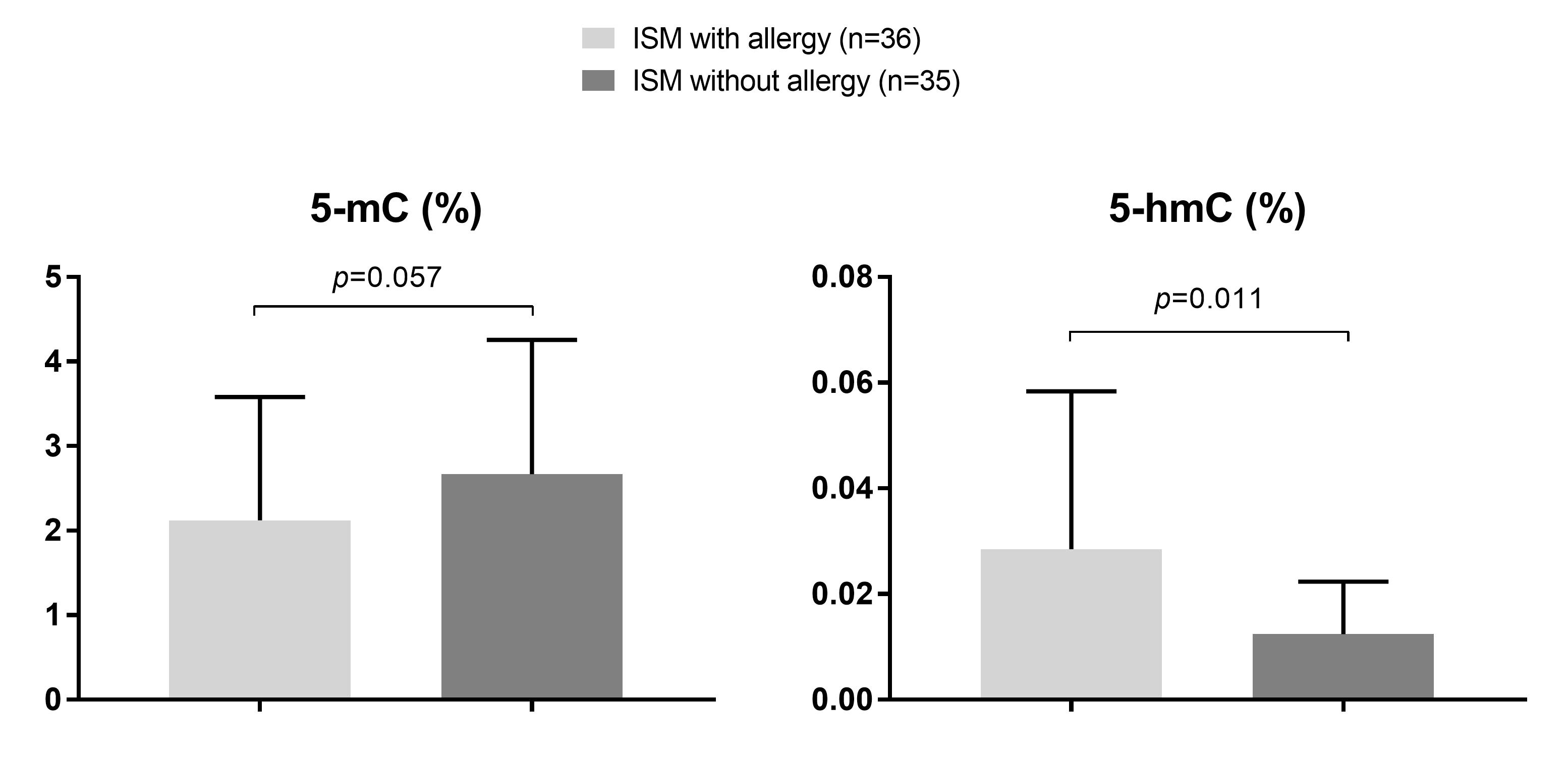

Supplement: Supplementary file 1 — Supplementary Material 1 [file CLT2-11-e12074-s001.jpg]
